# Emerging optogenetics technologies in biomedical applications

**DOI:** 10.1002/SMMD.20230026

**Published:** 2023-11-01

**Authors:** Haozhen Ren, Yi Cheng, Gaolin Wen, Jinglin Wang, Min Zhou

**Affiliations:** ^1^ Department of Hepatobiliary Surgery Hepatobiliary Institute Nanjing Drum Tower Hospital Medical School Nanjing University Nanjing China; ^2^ Department of Vascular Surgery The Affiliated Drum Tower Hospital of Nanjing University Medical School Nanjing China

**Keywords:** biomedical application, light delivery system, light‐sensitive protein, neurology, optogenetics

## Abstract

Optogenetics is a cutting‐edge technology that merges light control and genetics to achieve targeted control of tissue cells. Compared to traditional methods, optogenetics offers several advantages in terms of time and space precision, accuracy, and reduced damage to the research object. Currently, optogenetics is primarily used in pathway research, drug screening, gene expression regulation, and the stimulation of molecule release to treat various diseases. The selection of light‐sensitive proteins is the most crucial aspect of optogenetic technology; structural changes occur or downstream channels are activated to achieve signal transmission or factor release, allowing efficient and controllable disease treatment. In this review, we examine the extensive research conducted in the field of biomedicine concerning optogenetics, including the selection of light‐sensitive proteins, the study of carriers and delivery devices, and the application of disease treatment. Additionally, we offer critical insights and future implications of optogenetics in the realm of clinical medicine.

## Graphical abstract

Optogenetics is a sophisticated technology that employs a vector to transfect a photosensitive protein that responds to light to a specific target organ, cell, or gene to achieve directional regulation of cells or genes.

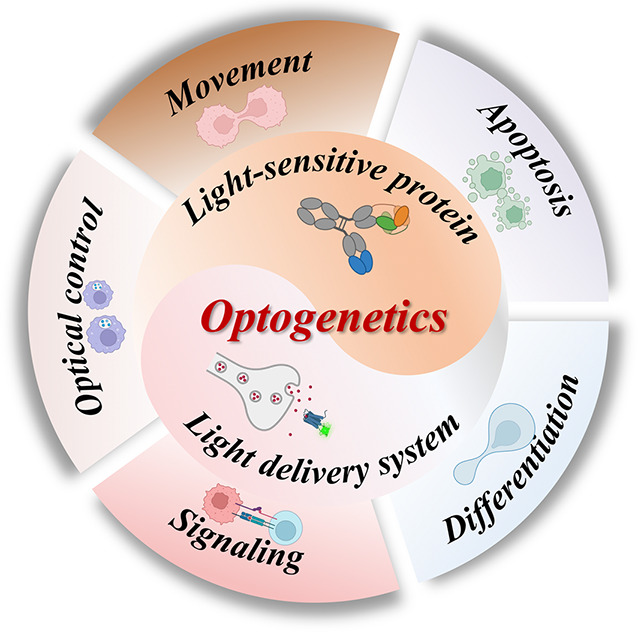

**Key points**

- Optetics employs vector transfection of photosensitive proteins to elicit light responses in specific target organs, cells, or genes, thus achieving targeted modulation of cells or genes and contributing to the organ regulation.
- Our review provides an overview of the emerging developments and biomedical applications of optogenetics.

## 1 INTRODUCTION

Optogenetics is an advanced technology that utilizes a vector to transfect a photosensitive protein, responsive to light, into specific target organs, cells, or genes, enabling precise regulation of cellular activities.^1^, ^2^ Examples of light regulation include the phototropism, light‐dependent growth response of plants, the visual perception of animals, and the photophilic movement of bacteria.^3^, ^4^, ^5^, ^6^, ^7^, ^8^ Optogenetics has several advantages over other tools for targeted regulation of cells, such as chemical methods, pure genetic methods, and hybridization methods.^9^, ^10^, ^11^ By employing light as a trigger, optogenetics offers meticulous temporal and spatial control over cellular expression, resulting in prompt responsiveness and high‐intensity manipulability.^12^, ^13^, ^14^, ^15^

Optogenetics is not only applicable to the rapid regulation of neuronal expression and neurological timing but also to the regulation of cell, tissue, and gene expression.^16^, ^17^ It can guide cells to move in a specific direction, control the activity of membrane receptors and downstream signaling proteins, regulate cell signaling pathways, induce tissue or cell actomyosin contractility, light‐trigger apoptosis, and control gene expression to determine cell differentiation fate, among other applications.^18^, ^19^, ^20^, ^21^, ^22^ This advantage is critical in the treatment of many acute clinical diseases, such as atrial fibrillation and epilepsy.^23^, ^24^ However, numerous challenges persist, including biocompatibility concerns, immune response issues, and other related obstacles.^25^, ^26^, ^27^, ^28^

**FIGURE 1:**
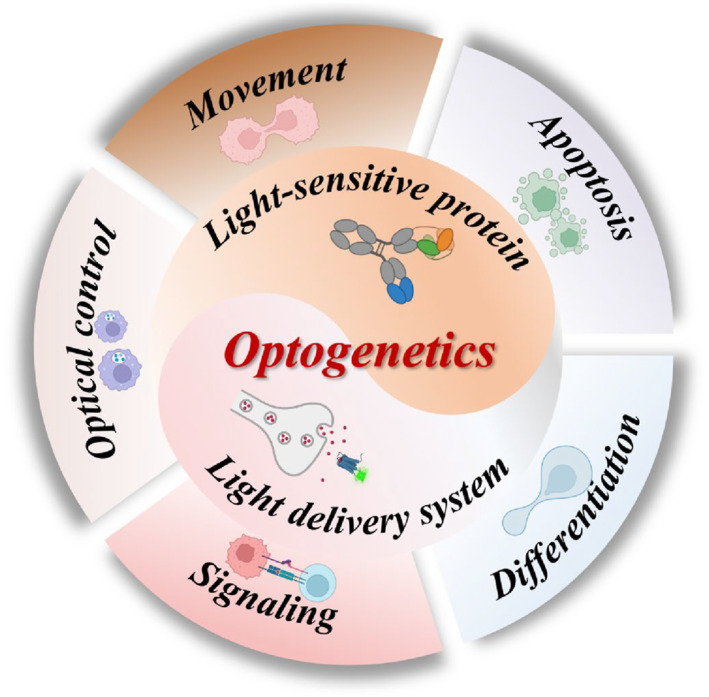
Key factors and application fields of optogenetics research.

In this article, we present an overview of optogenetics principles and the most recent research advances, covering the selection of light‐sensitive protein pathways and their potential clinical applications. We begin by providing a detailed exposition on the fundamental principles of optogenetics, including its research objectives, the selection criteria for light‐sensitive proteins, and the development of light‐sensitive protein carriers and delivery systems. Subsequently, we scrutinize the recent progress achieved in the application of optogenetics in biomedicine, with a particular emphasis on the innovative tools devised by optogenetics in the realms of neurology, metabolomics, oncology, genetics, and others (Figure 1). Lastly, we conclude by summarizing the challenges and prospects for future advancements in the clinical application of optogenetics.

## 2 CHARACTERISTICS AND SELECTION OF LIGHT‐SENSITIVE PROTEINS

**FIGURE 2:**
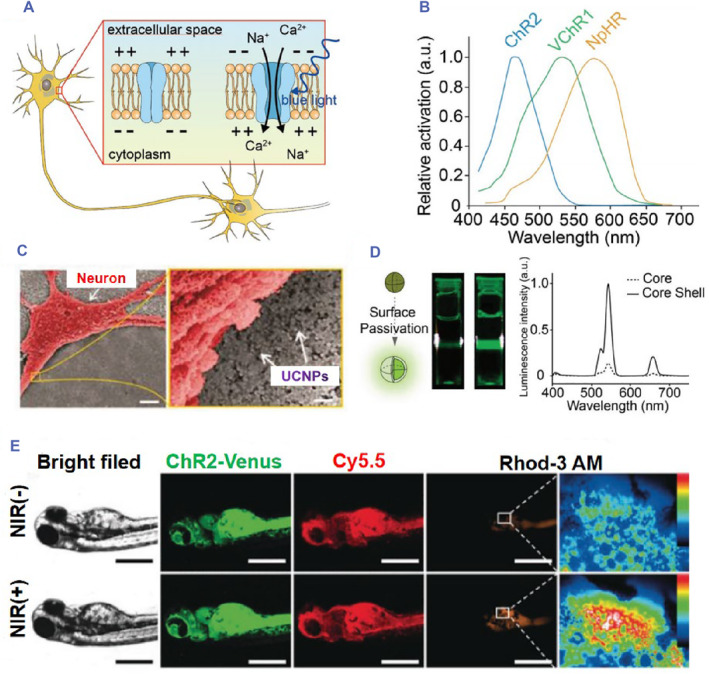
(A) The reaction of opsins to light stimulation. (B) Absorption spectra of opsins. (C) The picture of near‐infrared (NIR) ‐mediated optogenetics on neurons cultured onto the upconversion film. Scale bar: 5 μm (left), 200 nm (right). (D) A strategy for enhancing the upconversion emission intensity. (E) Fluorescence imaging of zebrafish incubated with Dibenzocyclooctyne (DBCO)/Cy5.5‐ up‐conversion nano‐phosphors (UCNPs) with and without NIR treatment. The Scale bar is 400 μm. Reproduced with permission.^29^ Copyright 2019, John Wiley and Sons.

The fundamental research objective of optogenetics is to identify an appropriate light‐sensitive protein and its corresponding carrier element based on the specific experimental requirements (Figure 2A–D).^29^ This is followed by the transfection of the viral vector into the target cells. However, given that light cannot penetrate deeply into the structures of the nervous system, it is necessary to surgically implant optical fibers that are connected to a laser (Figure 2E). Subsequently, a suitable illumination and expression time must be established. Hence, the primary distinguishing feature of optogenetics technology is the selection of photosensitive channels.^30^, ^31^, ^32^, ^33^ The selection of light‐sensitive proteins is a crucial aspect of optogenetics as it enables the transmission of light signals. These proteins are often derived from plants such as phytochromes, cryptochromes (CRYs), light‐oxygen voltage sensing (LOV), and ultraviolet resistance site 8 (UVR8) domain proteins, as well as from bacteria, archaea, algae, and higher animals. For example, channelrhodopsin (ChR2), halorhodopsin (NpHR), cyanobacterial pigments, CRYs, and other LOV domain proteins have been used in optogenetics research.^34^

Upon specific illumination, light‐sensitive proteins undergo conformational isomerization, aggregation, activation of downstream signaling channels, and other changes, thereby regulating cell, gene, and tissue function.^9^, ^35^, ^36^ There are various types of light‐sensitive proteins which differ in their mechanisms of action and can be classified based on their roles in either inhibiting or promoting cell or gene expression.^37^, ^38^ For instance, ChR2 mediates the influx of Na^+^ and Ca^2+^ and stimulates neuron excitation, while NpHR mediates the influx of Cl^−^ and inhibits neuronal activity.^39^, ^40^ Light‐sensitive proteins can also act through accumulation or dispersion to activate or inhibit pathways.^41^, ^42^ Based on their mechanism of action, photosensitive proteins can be classified into those that directly or indirectly generate effects. Examples of proteins that directly generate effects include photoactive yellow protein and LOV domain 2 (LOV2), which regulate the opening of ion channels, while visual proteins such as ChR2 directly control the transmission of neural signals. Indirectly generating effects, photosensitive proteins are covalently linked to the retina and transduce protein‐coupled reactions to light.^43^

The principles underlying the downstream signaling responses generated through protein‐protein, protein‐DNA, or protein‐lipid interactions have been extensively studied. Light can also lead to the polymerization, separation, and dissociation of photosensitive elements.^44^ Researchers need to select the appropriate photosensitive element based on the experimental requirement.^45^, ^46^, ^47^ Commonly used photosensitive channels include ChR2, which opens upon blue light induction and leads to cation influx and action potential generation, CRYs from Arabidopsis thaliana, which can induce polymerization using blue light and control the signaling pathway of polymerization transduction, and the LOV2 domain from oat, which undergoes conformational changes upon blue light induction to control target protein function.^18^

Light sensitivity is a key requirement for optogenetic studies, and it is important to consider the frequency of light required to activate the light‐sensitive proteins when selecting it for use in experiments.^48^, ^49^ Certain frequencies of light may have poor skin penetration, which limits the potential applications of some light‐sensitive proteins. For example, while cryptochromes 2 (CRY2) is sensitive to blue light, its poor skin penetration means that it may not be the optimal choice for some experiments. On the other hand, Phytochrome A (PhyA) pond to far‐red light, which has better skin penetration and can be used in in vivo research and clinical treatment.^50^, ^51^ Researchers must consider the specific experimental requirements when choosing a light‐sensitive protein. To overcome the disadvantage of poor light penetration, other techniques such as implanted optical fibers or light‐amplifying materials can be used. Some light‐sensitive proteins, such as UVR8, respond specifically to ultraviolet (UV) light, which can cause cytotoxicity, limiting their use to in vitro cell studies.^44^, ^52^, ^53^ However, there is a potential for UVR8‐based tools in the development of photoresponsive materials for drug delivery and biomaterial design.^54^, ^55^ As researchers continue to discover and explore new light‐sensitive proteins in nature, it will be important to identify their properties and potential applications.

## 3 OPTOGENETIC VECTOR DELIVERY AND OPTICAL FIBER IMPLANTATION

### 3.1 Viral vector

The selection of an appropriate photosensitive protein is only the first step in the development of optogenetic tools. Subsequent steps involve the efficient delivery and incorporation of the protein into target cells, genes or organs. Currently, viral vectors are the most widely used method for protein delivery.^56^, ^57^ These vectors include adenoviral vectors (AAV), retroviral vectors, lentiviral vectors, and others.^58^, ^59^, ^60^, ^61^, ^62^ Of these, AAV has proven to be a safe and effective vector for transduction.^63^, ^64^, ^65^ When used as a vector, recombinant AAV (rAAV) deletes all AAV protein sequences and adds the target protein. This approach retains only those sequences necessary for vector production or direct replication, reducing immunogenicity and cytotoxicity during in vivo delivery while maximizing the packaging capacity. Additionally, researchers have found that most natural AAVs are transduced centrally in the liver, while the capsids of AAV8 and AAV9 can target a variety of muscle types throughout the body. Therefore, rAAV gene therapy is mainly focused on the liver, striated muscle, and central nervous system (CNS) due to its high tissue targeting and transduction efficiency. Thus, rAAV represents an effective vector for therapeutic gene therapy and is currently the only viral vector approved for human use.

However, the rAAV vector is not without its limitations, and the issue of immune barriers must be addressed.^66^, ^67^, ^68^, ^69^, ^70^ Several approaches have been proposed to overcome this problem. One approach is to enhance the immune tolerance of the host organism, such as the use of B cell depletion and drug‐induced immune tolerance to achieve transient induction, or the incorporation of autologous cytokines, such as Treg cells, as adjuvants to modulate immune responses.^71^, ^72^, ^73^, ^74^, ^75^, ^76^ Another strategy is to optimize the rAAV capsid to evade innate immune surveillance, such as incorporating Toll‐like Receptor 9 (TLR9) inhibitory DNA sequences into the rAAV genome.^77^, ^78^ This innovative approach shows promise as a new strategy to address immune barriers and enhance the efficacy of rAAV‐based gene therapy.

**FIGURE 3:**
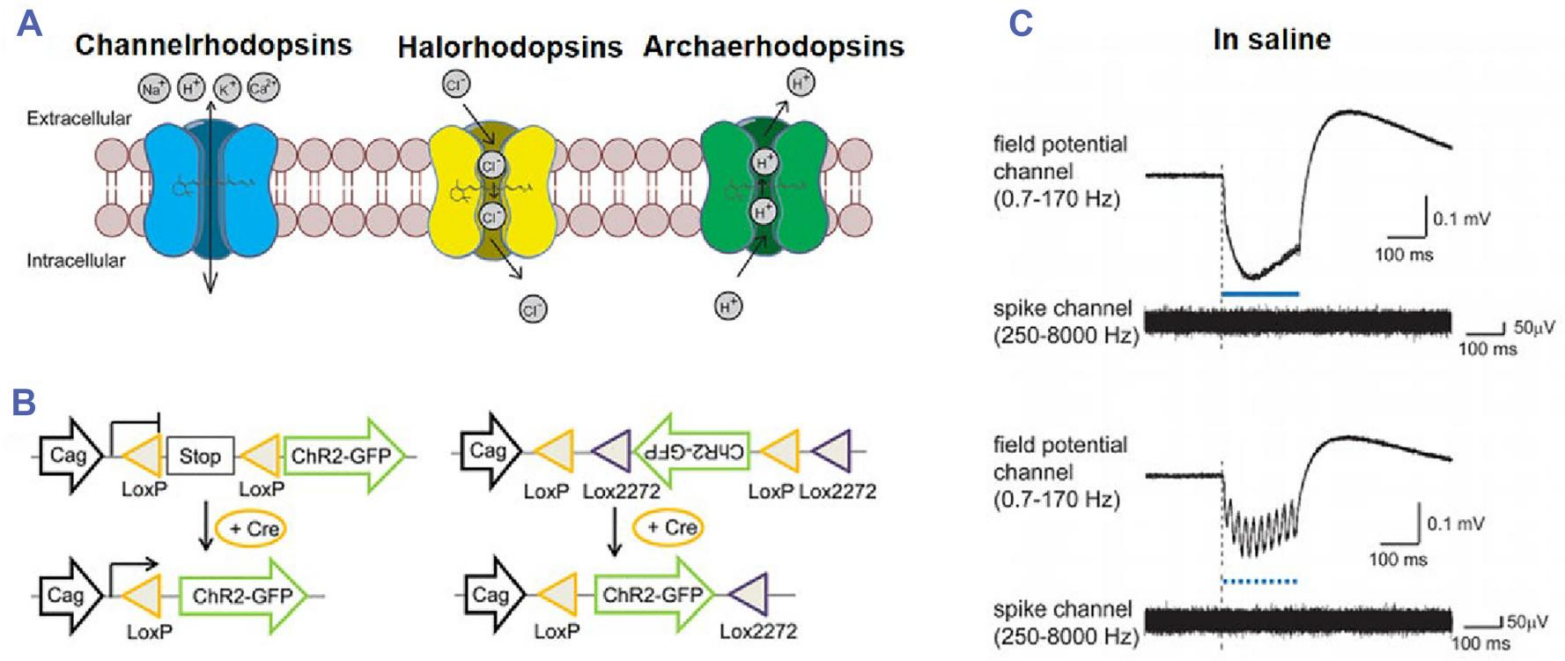
(A) Optogenetic molecular sensors. (B) Cell specific targeting. (C) Light‐mediated artifact under metal electrodes. Reproduced with permission.^80^ Copyright 2012, American Chemical Society.

One of the challenges of using rAAV vectors is their limited loading capacity, which is optimal for a genome under 5.0 kb. This requirement makes careful payload design necessary. However, the large size of many light‐sensitive proteins poses a challenge for viral vector transfection.^79^ To address this issue, researchers have developed non‐viral vectors such as cationic lipids, cationic polymers, nanoparticles, carbon nanotubes, gene guns, and calcium phosphate, which can potentially overcome the small loading capacity of viral vectors (Figure 3A–B). Although these non‐viral vectors can load DNA with higher molecular weight, their low in vivo conductivity remains a challenge that requires further research (Figure 3C).^80^

### 3.2 Light transmission problem

After resolving the loading issue of light‐sensitive proteins, the challenge remains to ensure that these proteins can sense light within the body, especially given the poor tissue penetration of certain wavelengths of light. One traditional approach has been to surgically implant an optical fiber; however, this method can lead to tissue damage and displacement due to animal movement.^81^, ^82^, ^83^, ^84^ To address this limitation, researchers have explored the use of far infrared light, which offers a deeper tissue penetration. For instance, Yang et al. have developed a PhyA‐based far‐red light‐mediated micro‐optical switch system (REDMAP), which has demonstrated various applications, including long‐term glycemic control through light modulation.^50^

Optogenetics has emerged as a powerful tool for studying biological processes with high spatiotemporal precision. However, the poor tissue penetration of some wavelengths of light poses a challenge for achieving efficient activation of light‐sensitive proteins in deeper tissues.^85^ To address this challenge, researchers have developed several strategies, such as combining nanomaterials with optogenetic structures to enable wireless optogenetics.^86^, ^87^, ^88^ For instance, UCNPs have been used to convert high‐penetrating, low‐energy near‐infrared light into high‐energy visible light or fluorescence, which is sufficient to excite voltage‐gated channels and generate action potentials.^89^, ^90^, ^91^, ^92^ Additionally, photothermal nanoparticles, such as gold nanoparticles (AuNPs), can convert light into heat energy and drive heat‐induced promoters to drive downstream signal expression.^93^, ^94^ Other nanoparticles, including mechanoluminescent and radioluminescent nanoparticles, have also been widely studied. These nanoparticles can be injected into the body, making this protocol biocompatible and an ideal solution for many optogenetic technologies.

## 4 APPLICATIONS OF OPTOGENETICS IN VARIOUS FIELDS

### 4.1 Nervous system

**FIGURE 4:**
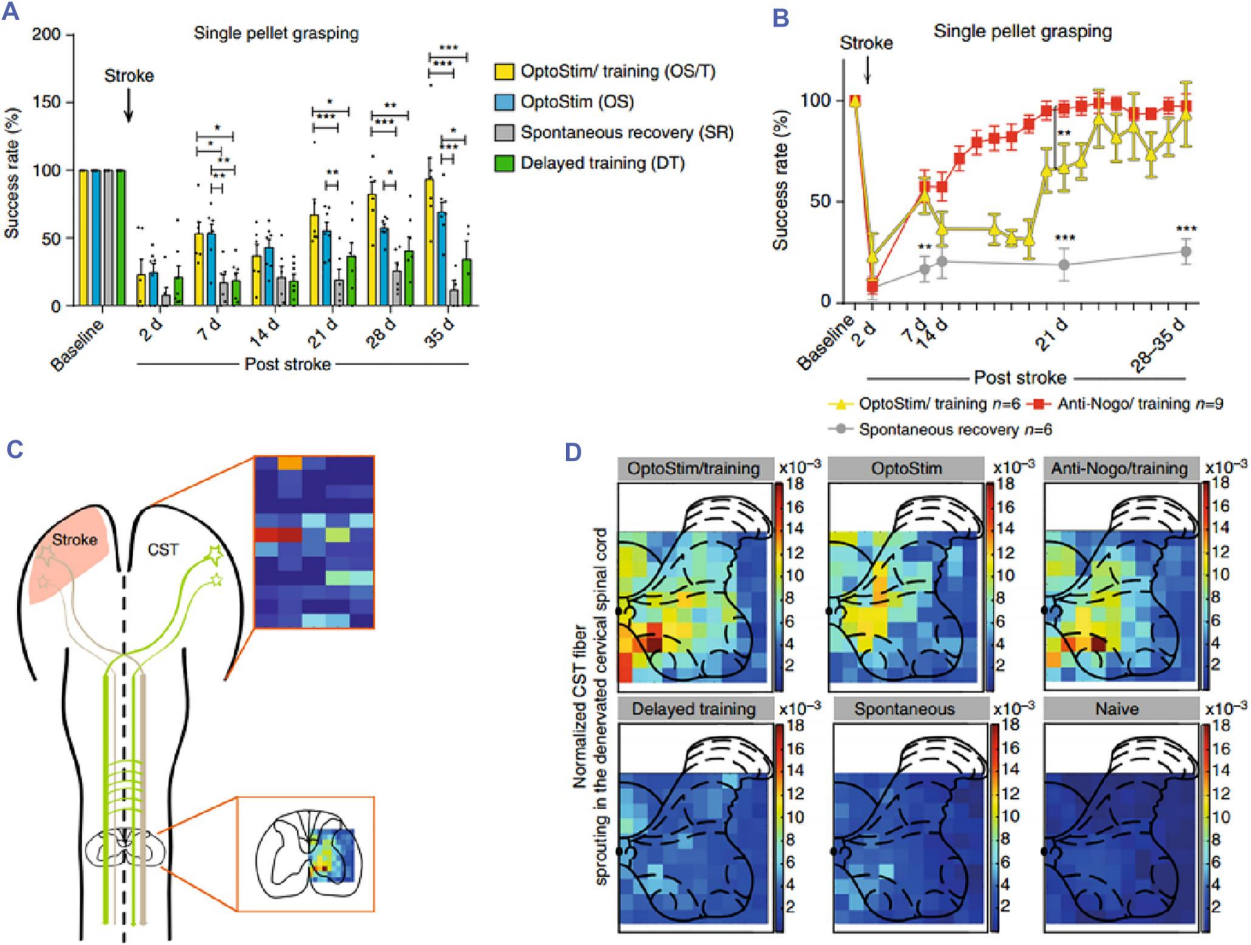
(A) The different success rates of the different groups. (B) The better results of the Anti‐Nogo/Training and OptoStim/Training groups than the stroke animals without treatment. (C) The scheme of results that localized functional reorganization and sprouting corticospinal tract (CST) fibers. (D) The heat maps showing the densities of CST fiber sprouting. Reproduced under terms of the CC‐BY license.^97^ Copyright 2017, The Authors, published by Springer Nature.

Optogenetics has found numerous applications in the field of neurology (Figure 4A),^95^, ^96^, ^97^, ^98^ including the investigation of animal behavior, optic nerve pathways, and treatment of neurological disorders such as Alzheimer's disease, stroke, pain management, and the role of peripheral nerves, among others (Figure 4B–D). In the past, various methods have been utilized to explore the role of the central nervous system, including electrical stimulation, magnetic therapy, light, among others.^96^, ^99^ However, these methods have significant disadvantages, such as poor specificity, large side effects, and uncontrollable effects. In contrast, optogenetic tools have overcome these limitations due to their precise targeting and time controllability, providing a promising approach for the precise study of central nervous circuits.^100^ For instance, activation of hypothalamic glutamatergic neurons through optogenetic tools has been found to induce sleep and wakefulness after general anesthesia, as reported by some researchers.^101^ Moreover, other researchers have found that direct light stimulation of memory trace cells in the hippocampus of mice with early Alzheimer's disease enables them to retrieve memories, even in transgenic mice with impaired long‐term memory.^95^ This approach provides a new direction for treating Alzheimer's disease, which was previously considered incurable.

**FIGURE 5:**
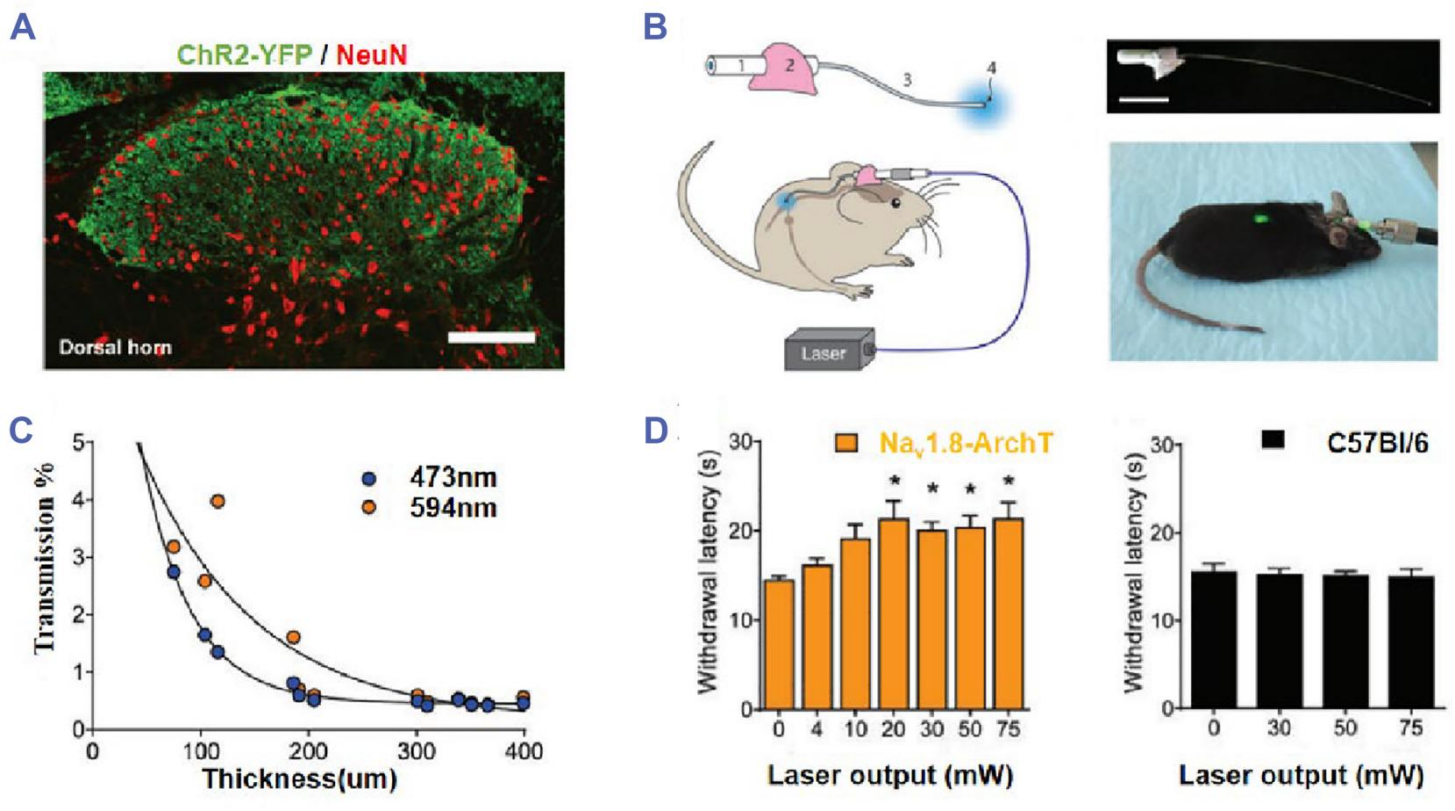
(A) Characterization of optogenetic mouse models. The scale bar is 100 μm. (B) Scheme of developing an epidural optic fiber implant. The scale bar is 1 cm. (C) Measurement of light transmission through spinal cord myelin of various thicknesses. (D) The lower withdrawal latencies to noxious thermal stimulation by delivery of orange light. Reproduced with permission.^103^ Copyright 2016, The Authors, published by SAGE Publications.

Optogenetics has emerged as a promising research avenue for analgesia in the field of neurology (Figure 5A–B). In recent years, numerous studies have explored optogenetics for analgesia research. For instance, a study utilized optical fibers and multi‐wavelength light stimulation to process epidural neurons in living animals, achieving nociceptive afferent inhibition in a mouse model by inhibiting neuronal activity (Figure 5C–D).^103^

**FIGURE 6:**
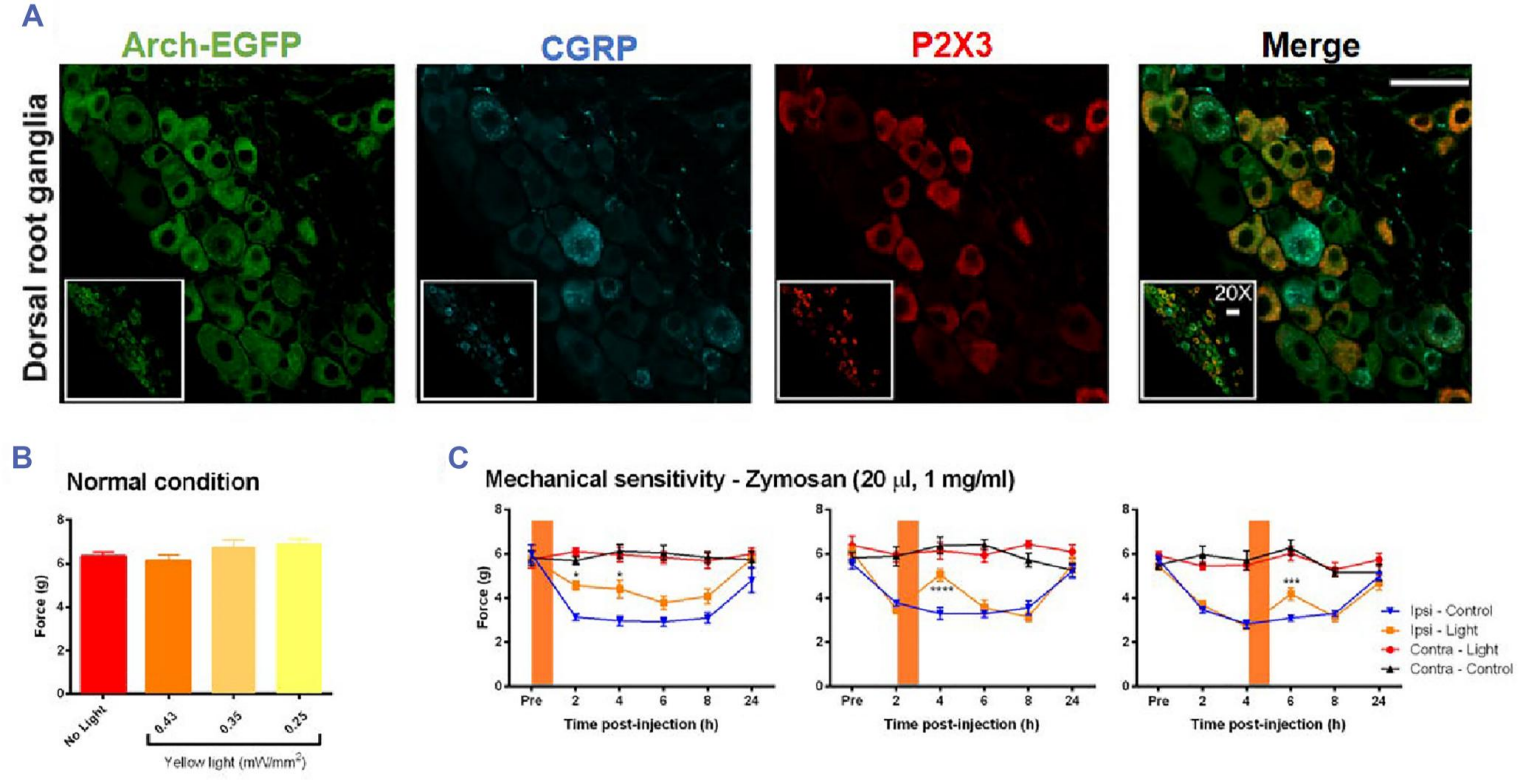
(A) Colocalization of Arch Arch‐ Enhanced Green Fluorescent Protein (EGFP) and Purinergic Recepto (P2X3) or Calcitonin gene related peptide (CGRP) showing its selective expression in nociceptors. The Scale bar is 50 μm. (B) Mechanical sensitivity of mice under different yellow light intensities. (C) Prolonged silencing caused poststimulation analgesia, resulting in zymosan‐mediated mechanical allodynia. Reproduced with permission.^104^ Copyright 2016, the Society for Neuroscience.

Furthermore, optical anesthesia has been achieved by using optical fibers to control the switch of the Arch‐3 proton pump on mouse neurons, not only inhibiting neuropathic pain from peripheral nerve afferents in mice but also significantly reducing inflammation (Figure 6).^104^ Over the past 2 years, an abundance of literature has reported on optogenetics research in analgesia. For example, Duan et al. utilized optogenetics to selectively activate or inhibit Lateral parabrachial nucleus (LPBN) glutamate neurons and γ‐aminobutyric acid (GABA) ergic neurons to observe pain‐like responses in normal mice and pathological pain mice.^105^ The results indicated that LPBN glutamate neurons' activity is not only crucial for the transmission of neuralgia and physiological pain but also a sufficient and necessary condition for neuralgia, while GABA ergic neurons play a key gating role in the occurrence and transmission of neuralgia.

**FIGURE 7:**
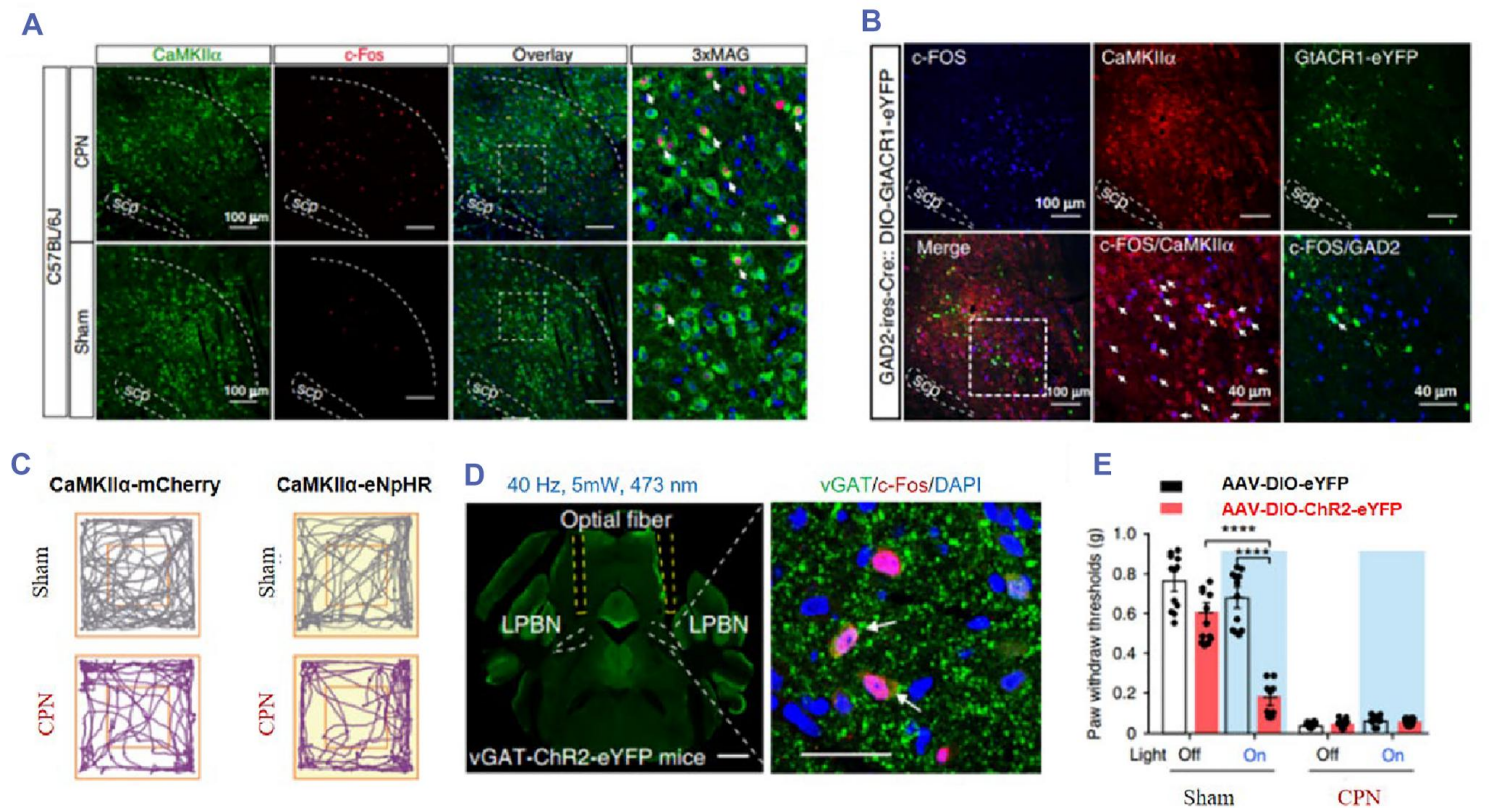
(A) Representative images of calmodulin dependent protein kinase IIα (CaMKIIα)‐positive neurons and Lateral parabrachial nucleus (LPBN). (B) Representative images of optogenetic inhibition. (C) Representative tracks of mice under different conditions. (D) Image showing optic fiber position under optogenetic stimulation. Scale bar: 1 mm (left), 30 μm (right). (E) The different results of the light of the LPBN. Reproduced under terms of the CC‐BY license.^105^ Copyright 2020, The Authors, published by Springer Nature.

This study is significant in deepening our understanding of the pathogenesis of neuralgia and providing a crucial new target for clinical intervention (Figure 7).^105^ Another study published utilized optogenetic tools to specifically inhibit or activate colonic epithelial cell activity and found that a reduction in epithelial activity may inhibit neuronal firing, thereby reducing visceral pain sensation.^106^ Optogenetic research in the field of neurology has yielded numerous insights, one of which is the use of optogenetic techniques for analgesia.^103^, ^107^, ^108^, ^109^ Several studies have been conducted in recent years, including the use of optical fibers and multi‐wavelength light stimulation to process epidural neurons in living animals to achieve nociceptive afferent inhibition and the use of optical fibers to control the switch of the Arch‐3 proton pump on mouse neurons to inhibit neuropathic pain from peripheral nerve afferents in mice and reduce inflammation.^104^

Optogenetics techniques can also be used in the peripheral nervous system, such as the use of optogenetic tools to surgically implant light‐emitting diodes around the bladder of mice to control the activation of specific bladder nerves and restore voiding function.^110^ Similarly, optogenetics has been used to prick the enteric nerve to promote intestinal peristalsis and gastric emptying by directly stimulating gastric smooth muscle cells.^111^ These advancements represent a promising new approach to treating a variety of neurological disorders.

Optogenetics is currently a rare technique in the field of neurology, as well as in other fields, due to the challenge of light penetration.^112^ Retinitis pigmentosa is a neurodegenerative eye disease that impairs the patient's vision, leading to difficulty distinguishing objects. Currently, there are no approved treatments other than gene replacement therapy for the early onset of the disease. Excitingly, the development of optogenetic goggles has pioneered a new, minimally invasive treatment.^113^, ^114^, ^115^, ^116^ In this therapy, an adeno‐associated virus vector containing rhodopsin is injected into the vitreous cavity of the patient's eye. The camera on the goggles converts the intensity of light into a light pulse, which is then projected onto the retina. The light‐sensitive protein responds to the light pulse, and the optic nerve is activated for imaging.^117^

### 4.2 Genetic and heart light system

Over the past decade, the heart light genetics have become a necessary tool for research on cardiac function and evaluation of different types of the function of the myocardial cells necessary tools.^118^, ^119^, ^120^, ^121^ The main research direction includes basic science research and treatment of heart diseases. In the realm of basic scientific researches, scholars have advanced the field by developing a comprehensive view of photoelectric measurement for the hearts of rats and a corresponding stimulation system.^122^ At the heart of this system lies a configuration of 294 optical fibers and 64 electrodes that span the entirety of the heart's ventricular surface and vessels in mice. This system allows for the precise customization of experiments to meet specific requirements. With the implementation of transgenic animal experiments involving the manipulation of gene expression in response to changes in light voltage, researchers have been able to record panoramic views of the optical and electrical activation maps of the heart. These studies have yielded critical insights into the genetics of the heart in mice, ultimately contributing to our broader understanding of cardiac function.

**FIGURE 8:**
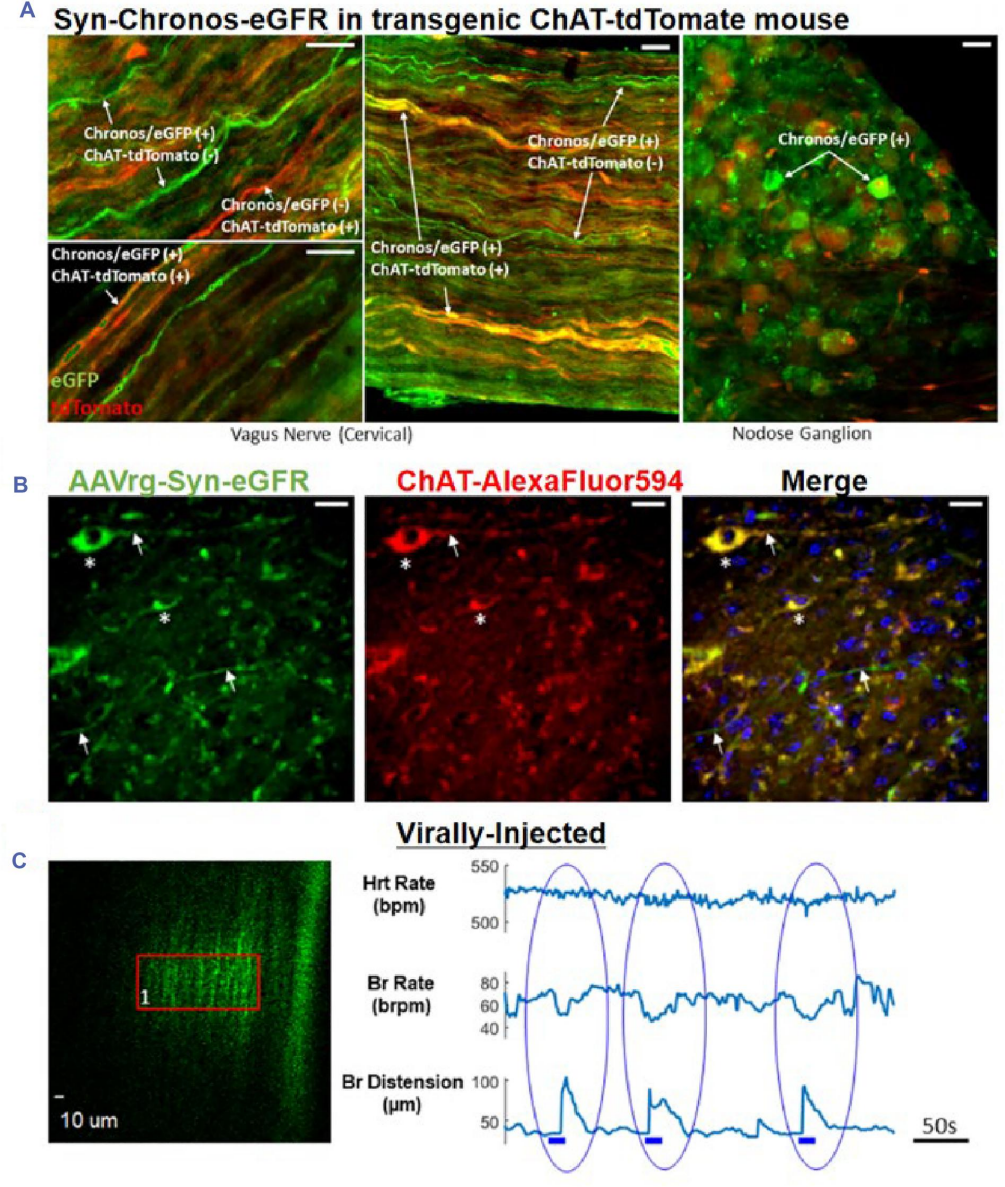
(A) The picture showing that the Syn‐Chronos‐Enhanced Green Fluorescent Protein (EGFP) was injected into Choline Acetyltransferase (ChAT)‐tdTomato transgenic mice. The Scale bar is 30 μm. (B) The pictures showing virally transduced neurons expressing EGFP within neurotensin (NTS). The Scale bar is 20 μm. (C) The robust response of the mouse under 920 nm photoexcitation. Reproduced under terms of the CC‐BY license.^127^ Copyright 2021, The Authors, published by Springer Nature.

Traditional treatments such as surgery, ablation, and medication, have been associated with a range of limitations, including irreversible damage to the heart tissue and adverse side effects. Optical genetic technology, however, is emerging as a highly precise technology that offers spatial and temporal control and is expected to replace some aspects of traditional treatment.^102^, ^123^, ^124^, ^125^, ^126^ Previous studies have demonstrated the feasibility of this technology in several types of heart diseases. For instance, cardiac pacemakers using light stimulation to control pulse rate have shown promising results in depolarization and repolarization. Painless defibrillation using light genetics has also exhibited advantages over traditional methods (Figure 8A).^127^ Additionally, cardiac resynchronization therapy utilizing diffuse light and protein expression has the potential to influence an unlimited number of remote sites. Furthermore, optical genetics have been shown to have neuromodulation (Figure 8B–C) and humoral regulation^128^ effects on cardiac function.^119^ These findings highlight the potential of optical genetic technology to revolutionize heart treatment and overcome the limitations of traditional approaches.

In the clinical management of chronic arrhythmia and heart failure due to different ventricular pacings, the electronic pacemaker has been the predominant treatment.^129^, ^130^, ^131^ However, electronic pacemakers have been associated with several drawbacks, including electrochemical reactions at the electrode tissue interface, invasive surgery, repeated battery replacement, infection risk, and equipment failure.^132^, ^133^, ^134^, ^135^, ^136^ Optogenetic technology, which involves transmitting various opsins to the heart through viral vectors and controlling heart rate through depolarization or repolarization stimulation, presents a promising alternative to electronic pacemakers.^137^, ^138^ Research has demonstrated that pacing the heart at multiple sites reduces activation time, thus leading to highly synchronized ventricular activation through the fusion of multiple wave fronts generated simultaneously by all chr2 expression sites.^139^ This finding suggests the feasibility of photogenetic biventricular pacing. Nonetheless, several challenges need to be addressed to optimize the use of photogenetic technology. These include the assurance of long‐term transgene expression, light transmission concerns, and the area and region of transduction, all of which significantly affect cardiac rhythm and contraction. For example, Nyns et al. used an adenovirus vector to transduce ReaChR to the rat heart and controlled heart rhythm through red light irradiation.^140^ However, this experiment was limited to the external body, and further research on light intensity, time, and region is needed.

The application of optogenetics in cardiac therapy holds great potential for regulating the cardiac rhythm and protecting the heart by modulating nerves and body fluids. The cardiac autonomic nervous system plays a critical role in adapting the heart to changes in contractility and the external environment. For instance, studies have demonstrated that vagus nerve stimulation can reduce intrinsic cardiac neuron remodeling and myocardial hypertrophy caused by chronic pressure load in guinea pigs, exerting a cardioprotective effect in the pressure and ischemia models.^141^

Despite the apparent regulatory effects of the nervous system on the heart, the circuit mechanisms involved are complex, and exploration under various conditions remains challenging.^142^ Photogenetic tools have revolutionized our ability to investigate the role of neural circuits in the brain and heart. Moreover, to enhance the survival rate of atrial fibrillation patients, researchers have explored the application of optogenetics to develop a defibrillation method that eliminates the need for high‐voltage shocks.^143^ This approach holds the promise of mitigating myocardial injury caused by high‐current electrical defibrillation. Nonetheless, several challenges must be addressed for this technology to be implemented in clinical practice, such as developing minimally invasive techniques and overcoming the issue of light penetration through the thick heart wall.^143^

### 4.3 Metabolism

**FIGURE 9:**
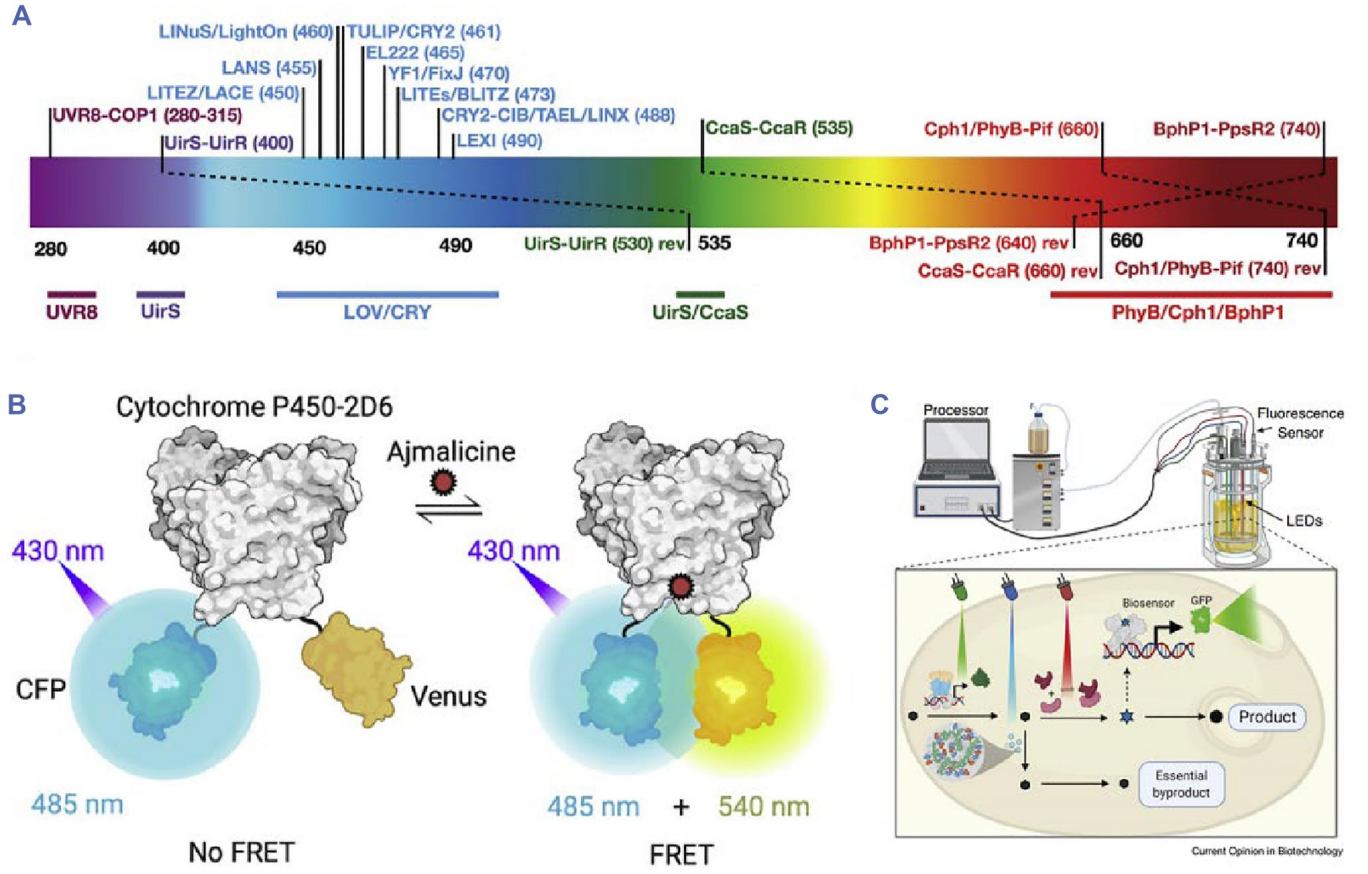
(A) The wavelengths of the optogenetic systems response span. (B) Under the connection of Ajmalicine, the fused cyan fluorescent protein (CFP) and Venus are more proximity to induce fluorescence resonance energy transfer (FRET). (C) General equipment of a metabolic cybergenetic system. Reproduced with permission.^145^ Copyright 2020, Elsevier.

Optogenetic systems have also found applications in metabolomics, including the regulation of glucagon secretion for blood glucose homeostasis,^144^ promotion of insulin expression,^50^ and development of microbial fermentation production (Figure 9).^145^ For instance, Zhou et al.^50^ have developed a miniature photoswitch control system based on the plant far‐infrared receptor PhyA, which has been applied in numerous ways. One of the applications involved implanting microcapsules containing light‐sensitive protein derived from human embryonic kidney cells (hck93) into diabetic rats through an adenovirus vector. Results showed that light‐treated diabetic rats experienced a significant increase in insulin secretion and blood sugar‐level control. This living cell‐based design can be utilized to express and secrete various therapeutic proteins for light‐controlled therapy for different diseases, allowing for long‐term and precisely controlled drug delivery.

Furthermore, optogenetics has been applied to microbial chemical production,^146^ utilizing periodic light pulses to regulate enzyme expression in the fermentation stage and enhance yield.^147^, ^148^ The researchers have introduced two primary systems, OptoEXP and OptoINVRT, which activate and inhibit various genomes through blue light irradiation, regulate endogenous and engineered metabolic pathways, and control the growth and production stages of fermentation to increase the target product yield.^149^ These systems offer unprecedented abilities for the manipulation, optimization, and automated fermentation of valuable product biosynthesis.

### 4.4 Cancer research

Immunotherapy has been hampered by the immunosuppressive microenvironment surrounding solid tumors.^150^, ^151^, ^152^, ^153^ While conventional methods can overcome tumor‐induced immunosuppression by administering immune‐enhancing factors systemically or directly into the tumor, their efficacy is limited by tumor growth and migration.^154^ Optogenetics holds promise in overcoming the limitations of tumor immunotherapy due to its spatial and temporal advantages.^155^, ^156^ For instance, ion channels can be activated to overcome Treg‐mediated immunosuppression.^157^ Optogenetics can also induce unprogrammed cell death in the tumor microenvironment and enhance leukocyte‐mediated tumor cell immunity.^158^, ^159^

Research has shown that the production of the inositol 1,4,5‐trisphosphate (IP_3_) is associated with the inhibition of cytotoxic T cells (CTL) killing function by Treg factors, indicating that the reduction of the IP_3_ concentration leads to a decrease in T cell receptor (TCR)‐dependent intracellular Ca^2+^ concentration.^160^, ^161^, ^162^ However, the process of regulating the calcium ion concentration through the IP3 protein pathway is complex and slow. Therefore, researchers have proposed the use of optogenetics to increase Ca^2+^ concentration, which can rapidly reduce the local Treg factor influence on CTL and relieve the immunosuppression of the microenvironment. Kim et al.^157^ transfected CTL cells with CatCh light‐sensitive protein, which mediates Ca2+ influx, and Ca^2+^ influx occurs under blue light irradiation, relieving the inhibitory effect of Treg factors. This conclusion was confirmed in melanoma mice, where the tumor in the experimental group was significantly smaller than that in the control group.

Unprogrammed cell death is characterized by its induction by inflammatory stimuli, transfer of functional proteins to cell membranes, membrane damage, cell swelling, calcium influx, and release of cellular contents, all of which can elicit immune responses. However, excessive necrosis can induce a range of inflammatory diseases, including sepsis. Recent research indicates that controlled induction of cell death through specific light stimulation can activate the immune system and alters the immunosuppressive state of the tumor microenvironment, offering a novel approach to cancer treatment.^158^ He et al. developed a novel optogenetic tool for inducing necroptosis and pyroptosis, which was named LiPOPs.^158^ In their study, CRYs and LOV2 proteins were employed as light‐sensitive proteins that responded to blue light irradiation, thus achieving a light‐controlled induction of apoptosis and pyroptosis. To overcome the problem of poor blue light transmittance, the research team utilized bioluminescence (NanoLOGS) and UCNPs as light‐emitting systems. To effectively control optogenetic tools using NanoLOGS technology, this study employed the newly developed substrate, Fluorofurimazine (FFz), which yielded more effective and durable bioluminescence effects. The study found that the substrate‐modified NanoLOGS had greater solubility, higher brightness, longer luminescence time, and good in vivo biocompatibility. This technique lays a foundation for investigating the role of non‐apoptotic cell death pathways in microbial infection and antitumor immunity.

In addition to considering the tumor immune microenvironment, tumor treatment can also control tumor occurrence by regulating the resting potential in the body. For the first time, researchers utilized photogenetics to manipulate the membrane potential mediated by *Xenopus laevis* tadpole ion channels in order to control oncogene‐induced tumors.^163^ They found that injection of mutated kras mRNA can induce tumor‐like structures. By expressing and activating ChR2 and Arch photosensitive proteins, the incidence of tumor formation can be significantly reduced by hyperpolarizing cells. This study introduces a novel method for tumor research and treatment.

## 5 CONCLUSION AND OUTLOOK

Over the past decade, optogenetics technology has emerged as a promising avenue for clinical precision treatment and research, offering potential solutions to the drawbacks of conventional drug or surgical therapies such as large trauma, slow effect, and significant side effects. In this review, we provide an overview of the three fundamental components of optogenetics, as well as recent advances in optogenetics research applications. We first discuss key aspects of optogenetic technology, including the selection of light‐sensitive proteins, vector design, and challenges associated with light transmission. Subsequently, we explore diverse applications of optogenetics across multiple fields, including neurology, oncology, genomics, and metabolomics.

Despite notable advancements in the depth and breadth of research, formulating appropriate protocols for clinical implementation remains a formidable task. Overcoming the challenge of limited light penetration is crucial for achieving widespread applicability within the human body. For instance, in the study of cardiac pathophysiology, myocardial injury or pressure can induce fibroblast differentiation. However, gene delivery of ChR2 blue light stimulation can emit directed and sustained light pulses, suppressing arrhythmias caused by fibrosis. Currently, the aforementioned research has mainly been conducted on cultured cardiac cells or computer models, with limited studies performed on beating animal models' hearts. Additionally, there are two crucial issues that still need to be addressed when applying optogenetics to the human heart: (1) Sufficiently high and abundant expression of photosensitive channel proteins in the myocardium and (2) Sufficient cardiac light transmission to activate these photosensitive channel proteins.

Current strategies involve substituting blue light‐responsive photosensitive proteins with far‐infrared counterparts possessing enhanced penetration capabilities, or employing light‐emitting diode (LED) lights, albeit with associated concealed risks. A promising approach involves the integration of nanomaterials and optogenetics, such as UCNPs, AuNPs, photothermal nanoparticles, mechanoluminescent nanoparticles, and radioluminescent nanoparticles. The substantial size of numerous light‐sensitive protein molecules presents obstacles in the delivery of standard gene therapy vectors, necessitating further investigation into carrier modification and adaptability to light‐sensitive proteins. Moreover, the method of illumination, encompassing factors such as intensity, interval time, illumination range, and pulse or continuous mode, profoundly influences treatment efficacy and necessitates comprehensive theoretical and experimental substantiation. We firmly believe that the application of optogenetics technology in biological research seeks to furnish more efficient, precise, and minimally invasive solutions for clinical disease treatment, ultimately augmenting patient survival rates and adaptability.

## AUTHOR CONTRIBUTIONS

Min Zhou and Jinglin Wang conceived the idea and revised the manuscript. Haozhen Ren conducted the investigation and wrote the manuscript. Yi Cheng and Gaolin Wen revised the manuscript.

## CONFLICT OF INTEREST STATEMENT

The authors declare that there are no competing interests.
